# An Investigation of Running Kinematics with Recovered Anterior Cruciate Ligament Reconstruction on a Treadmill and In-Field Using Inertial Measurement Units: A Preliminary Study

**DOI:** 10.3390/bioengineering11040404

**Published:** 2024-04-19

**Authors:** Matteo Hill, Pierre Kiesewetter, Thomas L. Milani, Christian Mitschke

**Affiliations:** Department of Human Locomotion, Chemnitz University of Technology, 09126 Chemnitz, Germany; cm-hill@outlook.de (M.H.); pierre.kiesewetter@hsw.tu-chemnitz.de (P.K.); thomas.milani@hsw.tu-chemnitz.de (T.L.M.)

**Keywords:** anterior cruciate ligament reconstruction, inertial measurement unit, running, triaxial peak tibial acceleration

## Abstract

Anterior cruciate ligament reconstruction (ACLR) may affect movement even years after surgery. The purpose of this study was to determine possible interlimb asymmetries due to ACLR when running on a treadmill and in field conditions, with the aim of contributing to the establishment of objective movement assessment in real-world settings; moreover, we aimed to gain knowledge on recovered ACLR as a biomechanical risk factor. Eight subjects with a history of unilateral ACLR 5.4 ± 2.8 years after surgery and eight healthy subjects ran 1 km on a treadmill and 1 km on a concrete track. The ground contact time and triaxial peak tibial accelerations were recorded using inertial measurement units. Interlimb differences within subjects were tested and compared between conditions. There were no significant differences between limbs in the ACLR subjects or in healthy runners for any of the chosen parameters on both running surfaces. However, peak tibial accelerations were higher during field running (*p*-values < 0.01; Cohen’s d effect sizes > 0.8), independent of health status. To minimize limb loading due to higher impacts during field running, this should be considered when choosing a running surface, especially in rehabilitation or when running with a minor injury or health issues.

## 1. Introduction

Anterior cruciate ligament (ACL) reconstruction (ACLR) following ACL rupture may have a long-term influence on the strength, proprioception, and movement kinematics of the affected limb, even after completing rehabilitation. This could raise the risk of a second ACL injury [1,2,3]. From a biomechanical perspective, common consequences appear to be changes in movement patterns, such as asymmetrical limb loading or avoidance patterns [4,5].

Various authors have described differences in movement execution during diverse motion patterns between the reconstructed and the healthy limb years after surgery [5,6,7]. The majority of studies on this issue were based on force plates and laboratory-restricted motion capture systems. The latter are considered the highest standard in describing and recording movement [8]. However, this means there is a lack of in-field investigations, which provide an environment closer to reality. In addition, marker-based motion capture systems require expert knowledge and long preparation times and are financially demanding, which make them difficult to use in clinical and everyday sports contexts [9]. As an alternative, inertial measurement units (IMUs) offer high mobility, flexible use in various environmental conditions, and easy application and are low-cost compared with motion capture systems, while delivering promising, quality data for objectively describing movement [9,10,11].

Running is a movement that is easy to execute, low-risk, and periodic and that appears in different forms in numerous sports. Therefore, it may be suitable to observe injury-induced changes in biomechanical parameters comparing in-field with laboratory conditions. According to a meta-analysis by Van Hooren et al., running on a motorized treadmill is comparable in large part regarding spatiotemporal, kinematic, and kinetic parameters [12]. Yet, they stated some differences among others in knee flexion and vertical loading. Johnson et al. [13] and Milner et al. [14] observed lower tibial shock values when running on a treadmill in contrast to running on surfaces outdoors, which may favor the former during early rehabilitation from an injury. This may be explained by a higher shock absorption of treadmills compared to other surfaces [15]. In general, running motions on various surfaces seem to be comparable with running on a treadmill, yet some biomechanical differences should be expected. Thus, the choice of surface may also have an influence on the development of injury-induced changes.

Vertical tibial acceleration (vPTA) is an established variable measurable using IMUs to analyze running biomechanics and, thus, lower limb loading for healthy subjects [16,17,18]. There are several studies using IMUs and focusing on the differences in tibial acceleration, comparing movements in the laboratory with movements in field conditions [13,14,19]. However, according to the authors’ knowledge, there is only one investigation examining lower limb accelerations with ACLR subjects’ IMUs during running, which was conducted in a laboratory [20]. Furthermore, there are only a few investigations on tibial accelerations in the medial–lateral and anterior–posterior directions in general [21], which may deliver a more complete perspective on knee loading and, consequently, provide important information when choosing a running surface [22]. Nevertheless, indirect comparisons may be drawn. Several authors have stated correlations between vPTAs and vertical ground reaction forces [21], such as between medial–lateral tibial accelerations (mlPTAs) and anterior–posterior tibial accelerations (apPTAs) compared with ground reaction forces in the corresponding directions [23].

As there is a lack of in-field investigations of running biomechanics after an injury, this study observed the long-term running kinematics of ACLR subjects after surgery using IMUs. The purpose was to determine possible interlimb asymmetries when running on a treadmill and in-field, with the aim of contributing to the establishment of objective movement assessment in real-world settings (i.e., outside of a laboratory setting), such as in sports and in clinical use. Further, it was intended to improve knowledge on the long-term influence of recovered ACLR on biomechanical behavior. We expected ACLR to influence interlimb symmetry in running kinematics (a1) characterized by lower tibial accelerations in the affected limb (a2). Furthermore, we hypothesized that in-field running would lead to higher tibial accelerations than running on a treadmill (b1). Lastly, interlimb differences for the ACLR subjects were expected to be more pronounced when running in-field than on a treadmill (b2).

## 2. Materials and Methods

### 2.1. Sample

A total of sixteen individuals participated in the study, consisting of eight subjects with a history of ACLR (4 male, 4 female; mean ± SD: age, 24.6 ± 3.1 years; body height, 172.1 ± 8.4 cm; body mass, 69.0 ± 7.8 kg; time after surgery, 5.4 ± 2.8 years) and eight healthy controls (4 male, 4 female; mean ± SD: age, 25.4 ± 3.9 years; body height, 171.8 ± 6.3 cm; body mass, 65.4 ± 9.9 kg). Individuals with acute pain or injury of the lower extremities and a history of knee surgery other than a one-time ACL surgery were not included in the study. Particular inclusion criteria for the experimental group were a one-time full rupture of the ACL and a full recovery from the following reconstruction. The surgery date had to be at least one and at most ten years before data acquisition. Participants were defined as fully recovered if they obtained permission for unrestricted physical activity on the part of a qualified medical professional. The study was conducted according to the guidelines of the Declaration of Helsinki and approved by the Institutional Ethics Committee of the Faculty of Behavioural and Social Sciences of the Chemnitz University of Technology (protocol code: 101541252). Informed consent was obtained from all subjects involved in the study.

### 2.2. Testing

**Figure 1:**
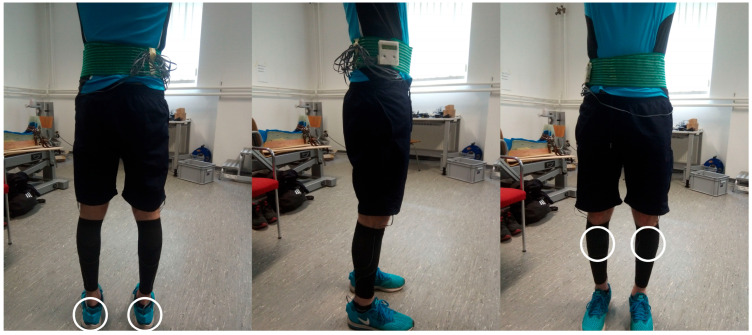
Full IMU setup. White circles indicate the positions of the placed sensors used for analysis.

Data acquisition was conducted at the biomechanical lab of the Chemnitz University of Technology on an approximately 1000 m long, straight, and flat concrete track close to the laboratory. After an initial measurement trial for calibration purposes and a 5 min warm-up and familiarization period on the treadmill, each subject ran 4 × 1 km in total: 2 × 1 km on the treadmill and 2 × 1 km on the concrete track. The possible sequences of the runs were equally distributed and randomly assigned to the participants. The distance of 1 km was selected based on Bräuer et al.’s study of biomechanical parameters during field runs [24]. The first run for each condition was excluded from further analysis as adaptational changes during the trials may have been expected. At least 5 min of break were given between single trials for recovery and transfer purposes. Running was performed at self-selected and constant speeds (for a minimum 9 km/h) on the treadmill and in the field runs. The running speed on the outdoor running track was constantly checked by the experimenter on a bicycle with a speedometer. Participants used their own running shoes for both running conditions. To ensure comparable results, control group subjects were motivated to run at similar speeds as experimental subjects. During trials, the runner’s motion was recorded using four small inertial measurement units (IMUs; ICM-20601, InvenSense, San Jose, CA, USA; weight: 4 g), which were attached with double adhesive and additional elastic tape to the rear part of the shoes and the shin bones (Figure 1) according to Kiesewetter et al. [25]. Each sensor unit consisted of a triaxial accelerometer (measurement range: ±353 m/s^2^) and a triaxial gyroscope (measurement range: ±4000°/s) to measure linear accelerations and angular velocities. The recording frequency was set to 2000 Hz. Each IMU was connected through wires to a data logger, which was worn on a belt around the waist. After trials, the rating of perceived exertion (RPE) and experienced pain in the knee were assessed.

### 2.3. Data Analysis

Raw acceleration and angular velocity signals were pre-processed with customized software and post-processed with MATLAB R2022b (MathWorksTM, Natick, MA, USA). Thereafter, the following spatiotemporal and kinematic parameters were obtained: ground contact time (GCT) and accelerations at initial ground contact, alongside the vertical (vPTAs), medial–lateral (mlPTAs), and anterior–posterior (apPTAs) axes of the tibia. To determine the initial ground contact, we applied a fourth-order high-pass filter at 80 Hz to the vertical compound of the feed’s acceleration signals [26]. The first positive peak of the signal at any stride was set as the corresponding initial contact [27]. The time point in which the acceleration of the foot-mounted sensor passed the 2 g threshold after the initial contact was set as the corresponding toe-off [28]. In order to identify peak tibial accelerations, a fourth-order Butterworth low-pass filter at 200 Hz was applied to the relevant signals. Positive peaks within any ground contact were determined as peak tibial accelerations for the equivalent direction. The mean values of each parameter throughout all the strides of one trial for each leg were calculated and used for further analysis.

### 2.4. Statistical Analysis

Statistical analysis was performed with MATLAB R2002b. Every data point’s distribution was checked visually by means of boxplots and statistically using the Kolmogorov–Smirnov test. Anthropometric data (age, weight, height) and running velocity were compared between groups using the Mann–Whitney *U* test, with the aim of guaranteeing matching groups. Limbs within groups were divided into ACLR limb (ACLRL) and healthy limb (HL) for the experimental group and left limb (LL) and right limb (RL) for the control group. Statistical testing for the GCT, vPTA, mlPTA, and apPTA was conducted within groups only, comparing the ACLRL and the HL as well as the LL and the RL for both conditions and limb-wise between conditions using the Wilcoxon signed-rank test. The alpha level was set to *p* < 0.05 and adjusted to *p* < 0.025 using Bonferroni correction due to multiple tests. Cohen’s d effect size was determined and interpreted for significant results. It was classified as follows: no (<0.2), small (≥0.2 and <0.5), medium (≥0.5 and <0.8), and large (≥0.8) effect [29]. Furthermore, correlations between interlimb differences and time after surgery were statistically investigated for the main parameters with Kendall’s tau test.

## 3. Results

### 3.1. Sample Characteristics

There were no statistically significant differences between the groups in terms of anthropometric data and running characteristics. The average running speed was 10.6 ± 1.2 km/h for the experimental group and 10.7 ± 1.1 km/h for the control group. Hamstring grafts were the most used graft type (four times). Two subjects received a patellar tendon graft, one a quadriceps tendon graft, and one a ligament repair.

### 3.2. Spatiotemporal Parameters

For the GCT, there were no significant differences at all between limbs in both groups for the treadmill condition, for the field condition, or between conditions comparing the same limbs (Table 1).

### 3.3. Peak Tibial Accelerations

**Figure 2:**
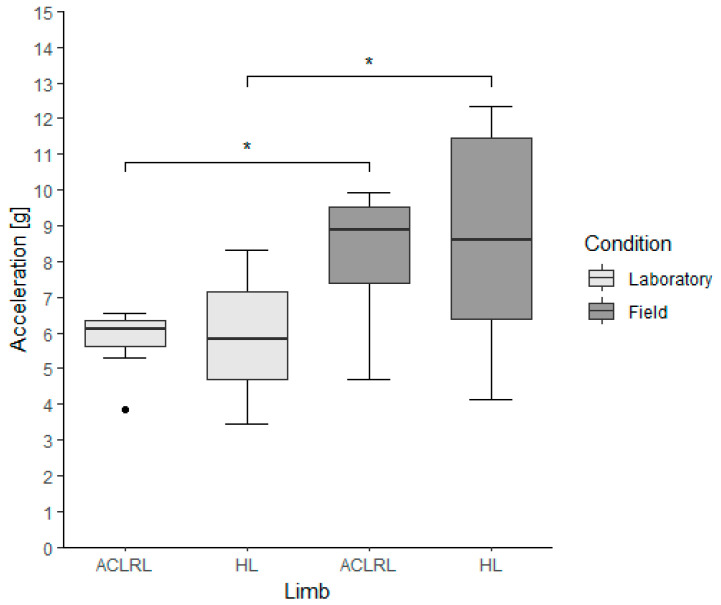
Vertical peak tibial acceleration values for the experimental group, divided by limb and running condition. ACLRL = anterior cruciate ligament reconstructed limb; HL = healthy limb; * *p* < 0.01; α = 0.025. ● represents outliers in the data sets. An outlier is defined as a value that is more than 1.5 times the interquartile range.

Regarding vPTA, there were no differences between limbs within the groups for both conditions (ACLR group, lab: ACLRL, 6.09 (0.72) g; HL, 5.81 (2.46) g; *p*-value, 0.945; field: ACLRL, 8.89 (2.11) g; HL, 8.61 (5.06) g; *p*-value, 0.641; accelerations in this section presented as median (interquartile range)). There were significantly higher vPTA values in the field condition for all limbs when comparing the same limbs between conditions (ACLR group, *p*-values (Cohen’s d): ACLRL, 0.008 (2.42); HL, 0.008 (6.9)) (Figure 2).

**Figure 3:**
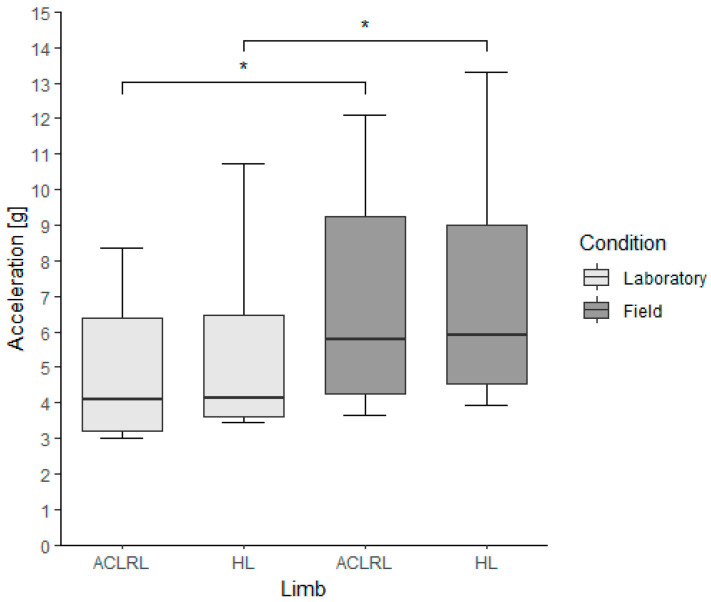
Medial–lateral peak tibial acceleration values for the experimental group, divided by limb and running condition. ACLRL = anterior cruciate ligament reconstructed limb; HL = healthy limb; * *p* < 0.01; α = 0.025.

In terms of the mlPTA, there were no differences between limbs within the groups for both conditions (ACLR group, lab: ACLRL, 4.08 (3.16) g; HL, 4.15 (2.86) g; *p*-value, 0.313; field: ACLRL, 5.76 (4.97) g; HL, 5.91 (4.46) g; *p*-value, 0.547). There were significantly higher mlPTA values in the field condition for the ACLRL, HL (ACLR group, *p*-values (Cohen’s d): ACLRL, 0.008 (2.16); HL, 0.008 (1.73)), and LL groups when comparing limbs between conditions (Figure 3).

**Figure 4:**
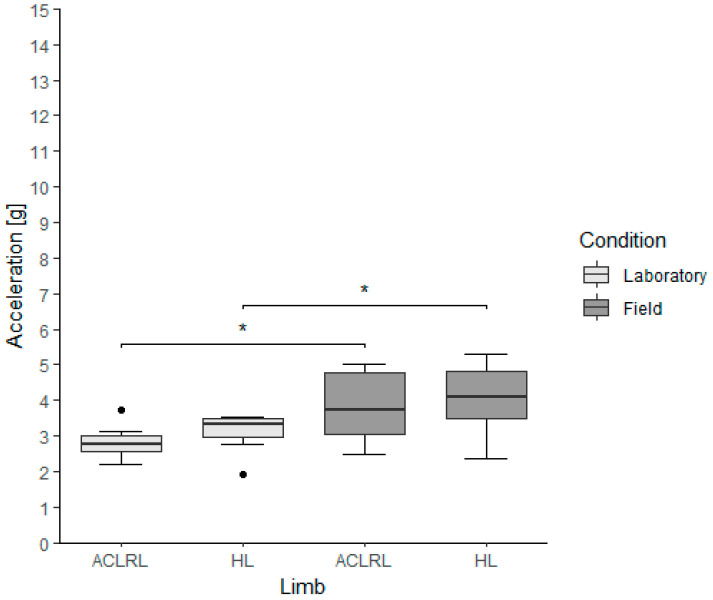
Anterior–posterior peak tibial acceleration values of the experimental group, divided by limb and running condition. ACLRL = anterior cruciate ligament reconstructed limb; HL = healthy limb; * *p* < 0.01; α = 0.025. ● represents outliers in the data sets. An outlier is defined as a value that is more than 1.5 times the interquartile range.

In terms of the apPTA, there were no differences between limbs within the groups for both conditions (ACLR group, lab: ACLRL, 2.77 (0.46) g; HL, 3.33 (0.54) g; *p*-value, 0.109; field: ACLRL, 3.72 (1.73) g; HL, 4.10 (1.31) g; *p*-value, 0.250). There were significantly higher apPTA values in the field condition for the ACLRL, HL (ACLR group, *p*-values (Cohen’s d): ACLRL, 0.008 (3.08); HL, 0.008 (0.81)), and LL groups when comparing limbs between conditions (Figure 4).

### 3.4. Time after Surgery

There were no significant correlations between interlimb differences and time after surgery for the GCT, vPTA, mlPTa and apPTA in the ACLR group. The results for the correlation coefficients were between τ = 0.074 and τ = −0.519.

## 4. Discussion

The aim of this study was to contribute to a better understanding of long-term changes in movement patterns after ACLR and to the establishment of objective movement analysis in real-world settings. The possible influence of an ACLR history on running kinematics was investigated using the example of running movements on a treadmill in the lab and on a concrete track outdoors. We hypothesized there would be an interlimb difference in running kinematics caused by the ACLR (a1) with lower tibial acceleration values in the affected limb (a2). Furthermore, we hypothesized that running on a concrete track would lead to higher tibial acceleration values than running on a treadmill for both groups (b1) and that interlimb differences for the ACLR subjects would be more pronounced during the former scenario (b2).

The results did not show any notable interlimb differences; hence, there was no long-term asymmetrical running behavior for ACLR subjects after surgery while running on a treadmill or on a concrete track. Interlimb symmetries based on the investigated variables in the experimental group were comparable to those in healthy individuals. Therefore, hypotheses a1, a2, and b2 were rejected. It seems that post-ACLR, individuals are able to adapt their running behavior in the long term to a sufficient level for various surface conditions. Nevertheless, tibial accelerations along all directions were significantly higher, with large effect sizes (Cohen’s d: 0.806–6.903) during the field condition, whereby hypothesis b1 was accepted. Yet, this was not the case for ground contact times.

The lack of interlimb differences in tibial accelerations during running in this study is supported by several authors. As tibial accelerations have been stated to correlate with the corresponding impact forces [21,23], there is evidence of no interlimb differences in vertical impact forces when running at least a year after surgery [30,31,32]. In contrast, Milandri et al. and Pamukoff et al. observed differences in impact loading between limbs [33,34]. Despite these conflicting results, there seems to be consensus in terms of interlimb differences in knee biomechanics. From a kinematic perspective, lower internal and external knee moments in the sagittal plane during walking and running in ACL-reconstructed limbs have been reported. In terms of kinematic parameters, lower knee peak flexion angles have been observed repeatedly [4,6,7]. Several explanations for this combination of lower knee loading and reduced peak knee flexion in the involved limb seem possible. On the one hand, there could be a reduced loading of the leg overall. If so, the reactional shock wave would be decreased and less attenuation through knee flexion and muscular activity would be required [35]. This would allow one to maintain a stiffer knee joint and consequently a stiffer leg. On the other hand, the actual strategy of a stiffer leg might be implemented beforehand through, e.g., avoidance patterns, while the overall limb loading is not reduced. This would lead to less muscular activation and may cause higher stress for passive structures, such as cartilage or bones [35]. To sum up, a stiffer leg pattern may be either a consequence of less limb loading overall or an adaptational strategy beforehand. All in all, it seems reasonable to expect differences in running behavior, yet there was no evidence of interlimb differences in tibial accelerations in this study. In addition, the literature focusing on tibial accelerations during running years after injury is scarce. It is important to add that there is no clear evidence of a progressive reduction in biomechanical alterations and interlimb asymmetries regarding the knee in the years following surgery when running or walking [36,37,38]. This coincides with the results of this study, as we could not find any correlation between time after surgery and interlimb differences.

In the ground contact times, we could not detect any differences between limbs and conditions overall. The GCT may be seen as an indirect spatiotemporal measure to describe running asymmetry [39]. In addition, there may exist a relationship between a short GCT and higher leg stiffness [40]. Concerning this, the obtained results for the GCT support the assumption that there is no long-term difference in running symmetry after ACLR.

The results in terms of higher tibial accelerations in the field conditions compared with the lab conditions are supported by the current literature [13,14,19]. As a concrete surface has stiffer material properties than a treadmill’s surface, the downward-moving leg will be slowed down after initial contact in a shorter amount of time and will therefore cause a higher reactional momentum [15,41]. Consequently, it seems reasonable to expect higher accelerations in the tibia after the initial contact. While most studies focus on vertical or resulting tibial accelerations, our results highlight that higher accelerations, and therefore higher knee loading, should be expected in all possible directions while running on concrete [13,19,21]. This may be considered when deciding on a running surface, especially in rehabilitation or when running with a minor injury or other health issues.

This study has some limitations that should be mentioned. Most importantly, the sample size was small, which diminished the possibility of detecting actual existing differences in running behavior and made the study prone to more extreme results. However, in this preliminary study, there was not any trend regarding interlimb differences at all, which may question the existence of such, even investigating a bigger sample size. Furthermore, it appears arguable that running is a suitable movement pattern to detect biomechanical abnormalities after an ACL injury. Running is not a movement that is usually considered to have a high risk of an ACL injury, such as cutting or landing [42,43]. The similarity in running symmetry for the ACL and healthy subjects in this study may be a consequence of good recovery and adaptational behavior from the ACL injury group. However, it seems reasonable to ask whether there was a difference in running behavior that was not detected by the chosen parameters. Therefore, it should be discussed whether tibial accelerations and the GCT are valid parameters to examine altered biomechanics in the long term after an injury. Furthermore, all subjects ran wearing their own shoes, both on the treadmill and during the field runs. It is important to note that the constitution of the shoes may lead to additional adaptations in running biomechanics. This influence may differ between surface conditions [44]. Finally, weather conditions during the field runs, such as wind or high temperatures, which could potentially impact the results, were not taken into further consideration.

Further research projects on tibial accelerations after injury should consider additional parameters, such as knee angle or knee moments. Moreover, there is still a lack of studies examining the influence of injuries on movement patterns using IMUs, especially in field conditions despite their advantages.

## 5. Conclusions

Our preliminary study did not show any interlimb differences during running on either the treadmill or on the concrete track in subjects years after ACLR. Yet, it cannot be concluded whether they adapted their running behavior to a sufficient level or whether actual existing differences could not be revealed due to the small sample size or the chosen parameters. In addition, triaxial tibial accelerations were higher during in-field running independent of health status. There has been a lack of research on cases of medial–lateral and anterior–posterior accelerations, which may provide a broader view on knee loading during running. Further research should focus on the influence of injuries on the biomechanics of movements in-field and should also consider more established parameters in this context. Overall, these are promising results that support the use of inertial measurement units to investigate biomechanical risk factors in real-world settings.

## Figures and Tables

**Table 1:** Median and interquartile range values for each limb, divided by condition. ACLRL = anterior cruciate ligament reconstructed limb; LL = left limb; HL = healthy limb; RL = right limb; MED = median; IQR = interquartile range; GCT = ground contact time; vPTA = vertical peak tibial acceleration; mlPTA = medial–lateral peak tibial acceleration; apPTA = anterior–posterior peak tibial acceleration. * Statistically significant differences for the same limb between conditions; *p* < 0.025.

	Laboratory		Field	
**Variable** (MED (IQR))				
Group	ACLRL	HL	ACLRL	HL
	LL	RL	LL	RL
**GCT (s)**				
ACLR	0.254 (0.038)	0.258 (0.033)	0.253 (0.034)	0.257 (0.034)
Controls	0.259 (0.021)	0.257 (0.022)	0.262 (0.018)	0.267 (0.026)
**vPTA (g)**				
ACLR	6.09 (0.72)	5.81 (2.46)	8.89 (2.11) *	8.61 (5.06) *
Controls	5.10 (1.64)	5.42 (1.63)	6.28 (0.68) *	6.49 (1.61) *
**mlPTA (g)**				
ACLR	4.08 (3.16)	4.15 (2.86)	5.76 (4.97) *	5.91 (4.46) *
Controls	3.71 (0.85)	3.62 (1.04)	4.93 (1.43) *	4.73 (2.09)
**apPTA (g)**				
ACLR	2.77 (0.46)	3.33 (0.54)	3.72 (1.73) *	4.10 (1.31) *
Controls	3.33 (1.27)	3.16 (0.89)	4.16 (1.57) *	3.79 (1.14)

## Data Availability

The data presented in this study are available on request from the corresponding author.
